# Reduced brain subcortical volumes in patients with glaucoma: a pilot neuroimaging study using the region-of-interest-based approach

**DOI:** 10.1186/s12883-022-02807-x

**Published:** 2022-07-25

**Authors:** Yae Won Ha, Heeseon Jang, Sang-Baek Koh, Young Noh, Seung-Koo Lee, Sang Won Seo, Jaelim Cho, Changsoo Kim

**Affiliations:** 1 Department of Public Health, Yonsei University College of Medicine, Seoul, Republic of Korea; 2 Department of Preventive Medicine, Yonsei University College of Medicine, Seoul, Republic of Korea; 3 Department of Preventive Medicine, Wonju College of Medicine, Yonsei University, Wonju, Republic of Korea; 4 Department of Neurology, Gil Medical Center, Gachon University College of Medicine, Incheon, Republic of Korea; 5 Department of Radiology, Severance Hospital, Yonsei University College of Medicine, Seoul, Republic of Korea; 6 Department of Neurology, Samsung Medical Center, Sungkyunkwan University School of Medicine, Seoul, Republic of Korea; 7 Institute of Human Complexity and Systems Science, Yonsei University, Incheon, Republic of Korea; 8 Institute for Environmental Research, Yonsei University College of Medicine, Seoul, Republic of Korea

**Keywords:** Glaucoma, Neuroimaging, Cortical thickness, Subcortical volume

## Abstract

**Background:**

While numerous neuroimaging studies have demonstrated that glaucoma is associated with smaller volumes of the visual cortices in the brain, only a few studies have linked glaucoma with brain structures beyond the visual cortices. Therefore, the objective of this study was to compare brain imaging markers and neuropsychological performance between individuals with and without glaucoma.

**Methods:**

We identified 64 individuals with glaucoma and randomly selected 128 age-, sex-, and education level-matched individuals without glaucoma from a community-based cohort. The study participants underwent 3 T brain magnetic resonance imaging and neuropsychological assessment battery. Regional cortical thickness and subcortical volume were estimated from the brain images of the participants. We used a linear mixed model after adjusting for potential confounding variables.

**Results:**

Cortical thickness in the occipital lobe was significantly smaller in individuals with glaucoma than in the matched individuals (β = − 0.04 mm, *P* = 0.014). This did not remain significant after adjusting for cardiovascular risk factors (β = − 0.02 mm, *P* = 0.67). Individuals with glaucoma had smaller volumes of the thalamus (β = − 212.8 mm^3^, *P* = 0.028), caudate (β = − 170.0 mm^3^, *P* = 0.029), putamen (β = − 151.4 mm^3^, *P* = 0.051), pallidum (β = − 103.6 mm^3^, *P* = 0.007), hippocampus (β = − 141.4 mm^3^, *P* = 0.026), and amygdala (β = − 87.9 mm^3^, *P* = 0.018) compared with those without glaucoma. Among neuropsychological battery tests, only the Stroop color reading test  score was significantly lower in individuals with glaucoma compared with those without glaucoma (β = − 0.44, *P* = 0.038).

**Conclusions:**

We found that glaucoma was associated with smaller volumes of the thalamus, caudate, putamen, pallidum, amygdala, and hippocampus.

**Supplementary Information:**

The online version contains supplementary material available at 10.1186/s12883-022-02807-x.

## Background

Glaucoma, one of the leading causes of blindness worldwide, damages the optic nerve and causes visual impairment [1, 2]. Glaucoma is strongly related to aging [3] and it is known that individuals with glaucoma are at high risk of brain health problems such as dementia, cognitive impairment, and depression [4–8]. Recent evidence suggests that glaucoma is an inflammatory neurodegenerative disease. Accordingly, aging and environmental stressors can induce mitochondrial dysfunction in retinal ganglion cells, and this process can increase the levels of inflammatory molecules (cytokines and chemokines), and consequently lead to glaucoma [9].

The effect of glaucoma on brain health has been reported by numerous studies using structural magnetic resonance imaging (MRI), which is useful in detecting neurodegeneration [10]. Studies have demonstrated that glaucoma patients have smaller visual cortex volumes in the brain (e.g., middle occipital gyrus, superior occipital gyrus, and precentral gyrus) [11–13]. However, only a few studies have investigated the link between glaucoma and changes in brain structures beyond visual cortices by utilizing brain MRI [14–16]. *Frezzotti* et al. reported that individuals with glaucoma had smaller frontoparietal cortex, hippocampus, and cerebellar cortex volumes compared with age-matched healthy individuals, using voxel-based morphometry [15]. *Wang* et al. conducted voxel-based and surface-based morphometry in 36 glaucoma patients and 20 healthy individuals, and found that the glaucoma patients had smaller volumes of the putamen, thalamus, and right hippocampus, as well as reduced cortical thicknesses in some small regions of the frontal, temporal, parietal, and occipital lobes [16]. Another study also found that glaucoma patients had decreased volumes of various small regions of the frontal, temporal, parietal, and occipital lobes compared with age- and sex-matched healthy individuals based on voxel-based morphometry [14]. These studies mainly used voxel-based morphometry, which allows the visualization of small brain regions associated with glaucoma, without predefining brain regions. By contrast, the region-of-interest (ROI)-based approach enables an easier clinical interpretation by defining clinically meaningful brain regions a priori. To the best of our knowledge, no study has used the ROI-based approach to investigate the linkage between glaucoma and brain structures beyond the visual cortices. Moreover, cortical thickness is known to be more sensitive to brain structural changes than volumetric measures [17, 18].

In this study, we aimed to compare ROI-based cortical thickness and subcortical volume on brain MRI between individuals with and without glaucoma. We also compared neuropsychological performance in these groups.

## Methods

### Study participants

This cross-sectional study was conducted as part of the Environmental Pollution-Induced Neurologic Effect (EPINEF) cohort study, described previously [19]. Briefly, the EPINEF cohort was established to determine risk factors for brain neurodegenerative diseases in the Republic of Korea. The cohort included adults (≥50 years) dwelling in two metropolitan cities (Seoul and Incheon) and two rural cities (Wonju and Pyeongchang). Those who had a history of stroke, dementia, or Parkinson’s disease were not eligible to participate. Baseline questionnaires included demographic information (age, sex, and socioeconomic status), lifestyle habits (smoking status and alcohol consumption), and their personal history of disease (cataract, glaucoma, hypertension, hyperlipidaemia, stroke, etc.). Participants also underwent physical measurement and blood sampling. A total of 3775 participants were enrolled in the cohort from August 2014 to March 2018. For this study, individuals who self-reported having a history of glaucoma were identified, and then their matched non-glaucoma individuals were selected. We conducted a 1:2 frequency matching based on age (±5 years), sex, education level (middle school graduation or not). Finally, 64 glaucoma group and 128 non-glaucoma group were included in this study.

### Acquisition and analysis of brain MRI

Using a standardized MRI protocol, 3D T1-magnetization images were obtained. Region-of-interest (ROI)-based cortical thickness and subcortical volume data were obtained through an automated brain image analysis tool (Inbrain®, MIDAS Information Technology Co., Ltd.), which is based on Freesurfer version 6.0.0 (http://surfer.nmr.mgh.harvard.edu/). Although segmentation errors were not manually checked, this automated brain image analysis tool improved the FreeSurfer reconstruction quality via deep learning in the procedures of brain extraction and white matter segmentation. In this procedure, we predefined brain regions as follows: frontal, temporal, parietal, occipital, and cingulate lobes. The predefined subcortical regions were the thalamus, caudate, putamen, palladium, hippocampus, amygdala, and nucleus accumbens. Intracranial volume was also estimated. The values for cortical thickness and subcortical volume in both the left and right hemispheres were averaged. Global cortical thickness was calculated by averaging the six regional cortical thickness values.

### Measurements of cognitive performance

Participants underwent the Mini-Mental state examination (MMSE) and a neuropsychological test (the Seoul Neuropsychological screening battery, SNSB) [20]. The neuropsychological battery assessment consists of the Rey Complex Figure Test (RCFT), Seoul Verbal Learning Test (SVLT), Korean version of Boston Naming Test (K-BNT), Controlled Oral Word Association Test (COWAT), Digit span test (DST), Korean Trail Making Test-Elderly (K-TMT-E), and Stroop test-color reading. Z-scores were adjusted for age, sex, and educational level in the calculation.

### Covariates

Marital status was defined as having a spouse or not. Personal history of diseases such as hypertension, angina or myocardial infarction, and diabetes mellitus was identified using self-reported questionnaires (“Have you ever been diagnosed by a doctor?”). Regarding lifestyle habits, smoking status was categorized as: never, former, and current smoker. Participants were also asked about current alcohol consumption status and categorized as current drinkers and former/non-drinkers. Systolic and diastolic blood pressure was classified as normal blood pressure, prehypertension, stage 1 hypertension, and stage 2 hypertension according to the Joint National Committee guideline 8 [21]. Body mass index (BMI) was categorized as underweight (< 18.5 kg/m^2^), normal weight (18.5-22.9 kg/m^2^), overweight (23. 0-24.9 kg/m^2^), and obese (≥25.0 kg/m^2^) according to the appropriate BMI for Asian populations [22]. Total cholesterol and fasting blood glucose level were obtained by analysing ≥ 12-hour fasting blood samples.

### Statistical analysis

We conducted Chi-squared tests for categorical variables and paired t-tests for continuous variables to investigate difference in characteristics between individuals with glaucoma and their age, sex, and education level-matched individuals. Differences in brain cortical thickness and subcortical volume between the above two groups were investigated using a linear mixed model (with unstructured covariance structure and restricted maximum likelihood), which considers both within-subject and between-subject correlations. In this study, the matched pair identifier was treated as a random effect. In the adjusted model, history of disease (hypertension, angina or myocardial infarction, and diabetes mellitus), lifestyle variables (smoking status and current alcohol consumption status), marital status, systolic and diastolic blood pressure, BMI, total cholesterol, fasting blood glucose, and intracranial volume were included. Differences in the MMSE and SNSB scores between the two groups were also investigated using the same model as in the analysis of brain cortical thickness. In the analysis of brain cortical thickness and subcortical volume, multiple comparisons were corrected using the false discovery rate method [23] and the Bonferroni-Holm method [24]. In addition, we conducted a post-hoc analysis considering that glaucoma can occur secondary to diabetes mellitus and hypertension. To ensure that cortical thickness and subcortical volume differences between individuals with and without glaucoma are not related to diabetes mellitus and hypertension, we stratified the study population on history of diabetes and hypertension and performed the same analyses. All statistical analyses were conducted using STATA version 14.0 (Stata Corp, USA) and SAS version 9.4 (SAS Institute, USA). A two-tailed test with a *P* < 0.05 was set as statistical significance.

## Results

The mean age of both the glaucoma group and the non-glaucoma group was 70 years old. Women accounted for 59.4% of the total population. The percentage of participants with hypertension was significantly higher in the glaucoma group compared to the non-glaucoma group (*P* < 0.001). Similarly, the percentage of participants with diabetes mellitus was higher in the glaucoma group than in the non-glaucoma group. The fasting blood glucose level was significantly higher in the participants with glaucoma than in those without glaucoma (*P* = 0.008) (Table 1).

**Table 1:** Demographic and clinical characteristics of the study population

Characteristics	Individuals with glaucoma (*N* = 64)	Individuals without glaucoma (*N* = 128)	p^a^
Age (years)	70.97 ± 5.37	70.57 ± 5.32	0.63
Sex			
Male	26 (40.6)	52 (40.6)	1.00
Female	38 (59.4)	76 (59.4)	
Education level			
< middle school	39 (60.9)	78 (60.9)	1.00
≥ middle school	25 (39.1)	50 (39.1)	
Marital status			
Married	51 (79.7)	97 (76.4)	0.61
Non-married	13 (20.3)	30 (23.6)	
Hypertension			
Yes	50 (78.1)	48 (37.5)	< 0.001
No	14 (21.9)	80 (62.5)	
Angina or myocardial infarction			
Yes	9 (14.1)	15 (11.7)	0.64
No	55 (85.9)	113 (88.3)	
Diabetes mellitus			
Yes	27 (42.2)	23 (18.0)	< 0.001
No	37 (57.8)	105 (82.0)	
Smoking status			
Current smoker	3 (4.7)	3 (2.3)	0.54
Former smoker	20 (31.3)	35 (27.3)	
Never smoker	41 (64.1)	90 (70.3)	
Alcohol consumption			
Yes	33 (51.6)	71 (55.9)	0.57
No	31 (48.4)	56 (44.1)	
Body mass index (kg/m^2^)			
< 18.5	0 (0.0)	1 (0.8)	0.68
18.5-22.9	18 (28.1)	33 (25.8)	
23.0-24.9	21 (32.8)	35 (27.3)	
≥ 25.0	25 (39.1)	59 (46.1)	
Systolic blood pressure (mmHg)			
< 120	19 (29.7)	31 (24.2)	0.74
120-139	28 (43.8)	62 (48.4)	
140-159	15 (23.4)	28 (21.9)	
≥ 160	2 (3.1)	7 (5.5)	
Diastolic blood pressure (mmHg)			
< 80	48 (75.0)	84 (65.6)	0.23
80-90	15 (23.4)	35 (27.3)	
90-100	0 (0.0)	7 (5.5)	
≥ 100	1 (1.6)	2 (1.6)	
Fasting blood glucose (mg/dL), mean (standard deviation)	109.94 ± 34.23	99.34 ± 20.66	0.008
Total cholesterol (mg/dL), mean (standard deviation)	186.23 ± 38.56	177.90 ± 33.44	0.13

^a^ Significance of difference between individuals with and without glaucoma; Chi-squared tests for categorical variables and paired t-tests for continuous variables were used.

The mean (standard deviation) cortical thickness of the occipital lobe was 1.88 mm (0.10) in the glaucoma group and 1.92 mm (0.09) in the non-glaucoma group. The glaucoma group had smaller subcortical volumes of the thalamus, caudate, pallidum, amygdala than the non-glaucoma group (Table 2).

**Table 2:** Differences in cortical thickness and subcortical volume between individuals with glaucoma and their age, sex, and educational level-matched individuals without glaucoma

		Individuals with glaucoma (*N* = 64)	Individuals without glaucoma (*N* = 128)	p^a^
		Mean ± standard deviation	Mean ± standard deviation	
Cortical thickness (mm)	Global	2.41 ± 0.09	2.43 ± 0.09	0.16
	Frontal lobe	2.46 ± 0.11	2.48 ± 0.11	0.39
	Parietal lobe	2.16 ± 0.12	2.20 ± 0.11	0.051
	Temporal lobe	2.70 ± 0.11	2.72 ± 0.12	0.17
	Occipital lobe	1.88 ± 0.10	1.92 ± 0.09	0.005
	Cingulate	2.45 ± 0.13	2.46 ± 0.14	0.58
	Insula	2.84 ± 0.13	2.84 ± 0.16	0.86
Subcortical volume (mm^3^)	Thalamus	6057.84 ± 644.70	6360.02 ± 675.99	0.003
	Caudate	3033.387 ± 447.86	3222.19 ± 510.70	0.013
	Putamen	4123.42 ± 466.39	4251.56 ± 531.30	0.10
	Pallidum	1729.90 ± 212.57	1817.86 ± 202.77	0.006
	Hippocampus	3646.42 ± 402.72	3757.53 ± 360.48	0.054
	Amygdala	1424.78 ± 196.56	1500.58 ± 207.17	0.016
	Nucleus accumbens	363.53 ± 69.87	380.01 ± 78.60	0.16

^a^ Significance of difference between individuals with and without glaucoma; calculated by paired t-test

The MMSE and SNSB scores were analysed and the glaucoma group had a significantly lower MMSE score than the non-glaucoma group (Table 3). In addition, the glaucoma group had lower average scores in SVLT recall, K-BNT, COWAT phonemic, and Stroop test-color readings compared to the non-glaucoma group.

**Table 3:** Differences in MMSE and SNSB test scores between individuals with glaucoma and their age, sex, and educational level-matched individuals without glaucoma

	Individuals with glaucoma (*N* = 64)	Individuals without glaucoma (*N* = 128)	p^a^
	Mean ± standard deviation	Mean ± standard deviation	
MMSE	24.95 ± 2.93	26.01 ± 3.07	0.016
SNSB			
SVLT recall total score	−0.39 ± 0.98	−0.31 ± 1.03	0.60
SVLT delayed recall	− 0.44 ± 1.17	− 0.46 ± 1.17	0.90
RCFT delayed recall	− 0.14 ± 0.95	− 0.33 ± 1.03	0.23
RCFT copy	−0.35 ± 0.99	− 0.82 ± 1.38	0.018
K-BNT	−0.45 ± 1.18	−0.32 ± 0.99	0.41
COWAT animal	−0.32 ± 1.09	−0.32 ± 0.90	1.00
COWAT phonemic	−0.57 ± 1.02	−0.46 ± 1.01	0.47
Stroop Test color reading	−0.73 ± 1.12	−0.30 ± 1.21	0.020
K-TMT-E Part B	−0.37 ± 1.70	−0.45 ± 1.69	0.79

*Abbreviations*: *MMSE* Mini-mental state examination, *SNSB* Seoul Neuropsychological Screening Battery, *SVLT* Seoul verbal learning test, *RCFT* Rey complex figure test, *K-BNT* Korean version of the Boston naming test, *COWAT* Controlled oral word association test, *K-TMT-E* Korean version of trail making test-Elderly

^a^ Significance of difference between individuals with and without glaucoma; calculated by paired t-test

In the unadjusted model, the glaucoma group had significantly reduced cortical thickness in the occipital lobe and reduced volumes of the thalamus, caudate, putamen, pallidum, hippocampus, and amygdala compared with the non-glaucoma group. In the adjusted model, the reduced occipital thickness in the glaucoma group did not remain significant. The reduced volumes of the subcortical structures remained significant, except for the putamen and nucleus accumbens (Table 4).

**Table 4:** Cortical thickness and subcortical volume in individuals with glaucoma compared to age, sex, and educational level-matched individuals without glaucoma

		Unadjusted model			Adjusted model		
		Beta	Standard error	p^*^	Beta	Standard error	p^*^
Cortical thickness (mm)	Global	−0.020	0.012	0.20	−0.011	0.014	0.75
	Frontal lobe	−0.015	0.015	0.46	−0.007	0.018	0.94
	Parietal lobe	−0.035	0.015	0.070	−0.021	0.017	0.67
	Temporal lobe	−0.024	0.015	0.20	−0.016	0.018	0.67
	Occipital lobe	−0.040	0.013	0.014	−0.016	0.015	0.67
	Cingulate	−0.011	0.018	0.62	−0.003	0.022	0.94
	Insula	0.004	0.021	0.86	0.002	0.024	0.94
Subcortical volume (mm^3^)	Thalamus	−300.45	73.97	0.004	−212.81	88.03	0.028
	Caudate	−187.61	63.20	0.007	−170.03	73.73	0.029
	Putamen	− 126.91	63.99	0.055	−151.41	75.03	0.051
	Pallidum	−87.45	26.36	0.004	−103.59	31.26	0.007
	Hippocampus	−110.42	47.58	0.028	−141.37	55.94	0.026
	Amygdala	−75.44	26.58	0.009	−87.94	31.42	0.018
	Nucleus accumbens	−16.32	10.17	0.11	−20.44	11.60	0.078

*Footnotes*. The adjusted model included the history of disease (hypertension, angina or myocardial infarction, and diabetes mellitus), lifestyle factors (smoking status and current alcohol consumption status), marital status, systolic blood pressure, diastolic blood pressure, body mass index, total cholesterol level, fasting blood glucose level, and intracranial volume. ^*^ *P*-values were corrected for multiple comparisons using the false discovery rate method

MMSE scores were lower in the glaucoma group than in the non-glaucoma group, but this difference showed borderline significance (*P* = 0.057) (Table 5). Scores for the total SVLT recall, K-BNT, COWAT phonemic, Stroop test-color reading, and K-TMT-E Part B tests were lower in the glaucoma group than in the non-glaucoma group, but only the Stroop test-color reading exhibited statistical significance (*P* = 0.04).

**Table 5:** MMSE and SNSB test scores in the glaucoma group compared to age, sex, and educational level-matched non-glaucoma group

	Unadjusted model			Adjusted model		
	Beta	Standard error	p	Beta	Standard error	p
MMSE	−1.13	0.45	0.013	− 1.02	0.54	0.057
SNSB						
SVLT recall total score	−0.074	0.15	0.62	−0.041	0.17	0.81
SVLT delayed recall	0.031	0.17	0.86	0.013	0.21	0.95
RCFT delayed recall	0.19	0.15	0.22	0.23	0.18	0.20
RCFT copy	0.47	0.20	0.017	0.48	0.23	0.039
K-BNT	−0.14	0.16	0.38	−0.050	0.19	0.79
COWAT animal	0.001	0.15	1.00	0.021	0.18	0.91
COWAT phonemic	−0.11	0.15	0.47	−0.14	0.18	0.45
Stroop Test color reading	−0.43	0.18	0.019	−0.44	0.21	0.038
K-TMT-E Part B	0.081	0.26	0.76	−0.067	0.29	0.82

*Footnotes*. The adjusted model included the history of disease (hypertension, angina or myocardial infarction, and diabetes mellitus), lifestyle factors (smoking status and current alcohol consumption status), marital status, systolic blood pressure, diastolic blood pressure, body mass index, total cholesterol level, and fasting blood glucose level

*Abbreviations*: *MMSE* Mini-mental state examination, *SNSB* Seoul Neuropsychological Screening Battery, *SVLT* Seoul verbal learning test, *RCFT* Rey complex figure test, *K-BNT* Korean version of Boston naming test, *COWAT* Controlled oral word association test, *K-TMT* Korean version of trail making test

After correcting for multiple comparisons using the Bonferroni-Holm method, only the associations with volumes of the pallidum and amygdala remained significant in the adjusted model (Supplementary Table 1). None of the differences in the associations of glaucoma with cortical thickness and subcortical volume between individuals with and without hypertension was statistically significant (Supplementary Table 2). None of the differences in the associations of glaucoma with cortical thickness and subcortical volume between individuals with and without diabetes mellitus was statistically significant, except insula (Supplementary Table 3).

## Discussion

This study investigated differences in the cortical thickness and subcortical volumes of the brain between the glaucoma group and their age, sex, and educational level-matched non-glaucoma group. We found that subcortical volumes of the thalamus, caudate, putamen, pallidum, hippocampus, and amygdala were significantly smaller in the glaucoma group when compared with the non-glaucoma group. This remained significant after adjusting for potential confounding variables. Although borderline insignificance was observed in the fully adjusted model, the MMSE score was significantly lower in individuals with glaucoma when compared with those without glaucoma. In addition, the Stroop test-color reading score was significantly lower in the individuals with glaucoma.

In this study, we observed that the volume of the brain regions associated with the cerebral limbic system was thinner in individuals with glaucoma compared with individuals without glaucoma. This finding is consistent with previous studies showing a decrease in hippocampus volume in individuals with glaucoma compared with individuals without glaucoma [15, 16]. A study with a small sample size (*n* = 25) also demonstrated that the hippocampus volume was smaller in individuals with glaucoma than in individuals without glaucoma, but the difference was not statistically significant [25]. Other studies have reported that the worsening of open-angle glaucoma was related to the atrophy of the hippocampus [26]. In addition to hippocampus atrophy, the volume of the amygdala was significantly reduced in individuals with glaucoma in our study, consistent with a previous report [25]. Given that the hippocampus and amygdala are responsible for memory and emotion [27, 28], the reduced volumes of the hippocampus and amygdala in individuals with glaucoma could lead to memory impairment and mood disorders. This notion is in line with epidemiological studies showing that individuals with glaucoma are at a higher risk for Alzheimer’s disease [29, 30] and depression [31–33]. Additionally, volumes of the basal ganglia (thalamus, caudate, putamen, and pallidum) were reduced in individuals with glaucoma compared to those without glaucoma in our study. This is consistent with previous studies demonstrating smaller volumes of the thalamus [16] and the caudate [34] in individuals with glaucoma compared to those without glaucoma.

In our study, individuals with glaucoma had a significantly reduced occipital thickness, but this reduction did not remain significant after adjusting for covariates, including hypertension and diabetes mellitus. Previous studies have shown mixed results regarding the association between glaucoma and the occipital lobe thickness. Some studies have reported smaller occipital lobe volumes in individuals with primary open-angle glaucoma compared with those without glaucoma [14, 15, 34] whereas another study showed a greater occipital lobe volume in glaucoma patients [35]. Future studies are required to investigate the association between glaucoma and the occipital lobe.

There are biological mechanisms that may explain the linkage between glaucoma and brain volume reduction (or neurodegeneration). One of the mechanisms is that glaucoma and neurodegenerative diseases (mainly Alzheimer’s disease) share common pathological features. The development of Alzheimer’s disease is caused by abnormal protein (amyloid beta and hyperphosphorylated tau) accumulation in the brain. An eye tissue analysis in mice with glaucoma demonstrated that an increase in amyloid beta protein was significantly associated with apoptosis of retinal nerve cells [36]. Another study found that tau transgenic (knockout) mice had hyperphosphorylated tau proteins in the nerve fibre layer of retinal ganglion cells [37]. An alternative mechanism is neuroinflammation in retinal ganglion cells. Internal and external stressors can induce mitochondrial dysfunction in retinal ganglion cells via increased oxidative stress and activation of inflammatory processes (e.g., proinflammatory cytokine and chemokine production, blood-retinal barrier damage, T-cell migration and activation [9]). In order to better understand the mechanism that underlies the association between glaucoma and brain structures, it would be desirable to relate morphological and functional abnormalities in glaucoma patients to brain structural changes. Future studies may need to assess the burden of amyloid beta and tau protein as well as the level of inflammatory markers in glaucoma patients and investigate whether these changes affect brain cortical thickness and subcortical volume.

This study has several limitations. First, because of the cross-sectional nature, it is not possible to infer a temporal relationship between glaucoma and the brain volume reduction. Future studies are needed to investigate the temporal relationship by following brain MRI markers. Second, there is a possibility of unmeasured confounding variables. For example, our data did not collect information on the type or duration of glaucoma in the participants. However, there is a paucity of evidence suggesting differences in brain MRI markers between the different types of glaucoma. Only one study has reported a decreased occipital lobe volume in individuals with high tension glaucoma versus normal tension glaucoma [38]. Similarly, given that glaucoma can occur secondary to diabetes mellitus [39, 40], subcortical volume reductions that we found to be associated with glaucoma in this study could also be attributed to diabetes mellitus. To attempt to address this limitation related to glaucoma secondary to diabetes, we additionally conducted the analyses stratified by history of diabetes and hypertension. Differences in the associations of glaucoma with cortical thickness and subcortical volume between individuals with and without history of hypertension were not statistically significant (Supplementary Table 2). Differences in the associations of glaucoma with cortical thickness and subcortical volume between individuals with and without history of diabetes were not statistically significant, except insula (Supplementary Table 3).

## Conclusions

In conclusion, our study showed that individuals with glaucoma had smaller volumes of the thalamus, caudate, putamen, pallidum, amygdala, and hippocampus compared with their age-, sex-, and education level-matched individuals without glaucoma, after adjusting for potential confounders. These findings add to the existing literature on the association between glaucoma and brain structures, beyond the visual cortices. Large-scale prospective studies are needed to further confirm this association.

## Supplementary Information

**Additional file 1: Supplementary Table 1.** Cortical thickness and subcortical volume in individuals with glaucoma compared to age, sex, and educational level-matched individuals without glaucoma (corrected for multiple comparisons using the Bonferroni-Holm method). **Supplementary Table 2.** Association of glaucoma with cortical thickness and subcortical volume, stratified by history of hypertension. **Supplementary Table 3.** Association of glaucoma with cortical thickness and subcortical volume, stratified by history of diabetes mellitus.

## Data Availability

The datasets generated and/or analyzed during the current study are not publicly available to ensure privacy protection of the participants, but are available from the corresponding author on reasonable request.

## Abbreviations

BMI: Body mass index
COWAT: Controlled Oral Word Association Test
DST: Digit span test
EPINEF: Environmental Pollution-Induced Neurologic Effect
K-BNT: Korean version of Boston Naming Test
K-TMT-E: Korean Trail Making Test-Elderly
MMSE: Mini-Mental state examination
RCFT: Rey Complex Figure Test
ROI: Region-of-interest
SNSB: Seoul Neuropsychological screening battery
SVLT: Seoul Verbal Learning Test

## References

[1] Bourne RR, Stevens GA, White RA, Smith JL, Flaxman SR, Price H (2013). Causes of vision loss worldwide, 1990-2010: a systematic analysis. Lancet Glob Health..

[2] Quigley HA, Broman AT (2006). The number of people with glaucoma worldwide in 2010 and 2020. Br J Ophthalmol..

[3] Guedes G, Tsai JC, Loewen NA (2011). Glaucoma and aging. Curr Aging Sci..

[4] Xiao Z, Wu W, Zhao Q, Liang X, Luo J, Ding D (2020). Association of Glaucoma and Cataract with Incident Dementia: A 5-Year Follow-Up in the Shanghai Aging Study. J Alzheimers Dis..

[5] Mancino R, Martucci A, Cesareo M, Giannini C, Corasaniti MT, Bagetta G (2018). Glaucoma and Alzheimer Disease: One Age-Related Neurodegenerative Disease of the Brain. Curr Neuropharmacol..

[6] Mullany S, Xiao L, Qassim A, Marshall H, Gharahkhani P, MacGregor S, et al. Normal-tension glaucoma is associated with cognitive impairment. Br J Ophthalmol. 2021. 10.1136/bjophthalmol-2020-317,461. 10.1136/bjophthalmol-2020-317461 PMC9234418 33781990

[7] Su CW, Lin CC, Kao CH, Chen HY (2016). Association Between Glaucoma and the Risk of Dementia. Medicine (Baltimore)..

[8] Vidal KS, Suemoto CK, Moreno AB, Duncan B, Schmidt MI, Maestri M (2020). Association between cognitive performance and self-reported glaucoma in middle-aged and older adults: a cross-sectional analysis of ELSA-Brasil. Braz J Med Biol Res..

[9] Duarte JN (2021). Neuroinflammatory Mechanisms of Mitochondrial Dysfunction and Neurodegeneration in Glaucoma. J Ophthalmol..

[10] Young PNE, Estarellas M, Coomans E, Srikrishna M, Beaumont H, Maass A, Venkataraman AV, Lissaman R, Jiménez D, Betts MJ, McGlinchey E, Berron D, O’Connor A, Fox NC, Pereira JB, Jagust W, Carter SF, Paterson RW, Schöll M (2020). Imaging biomarkers in neurodegeneration: current and future practices. Alzheimers Res Ther..

[11] Dai H, Morelli JN, Ai F, Yin D, Hu C, Xu D (2013). Resting-state functional MRI: functional connectivity analysis of the visual cortex in primary open-angle glaucoma patients. Hum Brain Mapp..

[12] Fukuda M, Omodaka K, Tatewaki Y, Himori N, Matsudaira I, Nishiguchi KM (2018). Quantitative MRI evaluation of glaucomatous changes in the visual pathway. PLoS One..

[13] Nuzzi R, Dallorto L, Rolle T (2018). Changes of Visual Pathway and Brain Connectivity in Glaucoma: A Systematic Review. Front Neurosci..

[14] Chen WW, Wang N, Cai S, Fang Z, Yu M, Wu Q (2013). Structural brain abnormalities in patients with primary open-angle glaucoma: a study with 3 T MR imaging. Invest Ophthalmol Vis Sci..

[15] Frezzotti P, Giorgio A, Motolese I, De Leucio A, Iester M, Motolese E (2014). Structural and functional brain changes beyond visual system in patients with advanced glaucoma. PLoS One..

[16] Wang Y, Wang X, Zhou J, Qiu J, Yan T, Xie Y (2020). Brain morphological alterations of cerebral cortex and subcortical nuclei in high-tension glaucoma brain and its associations with intraocular pressure. Neuroradiology..

[17] Burggren AC, Zeineh MM, Ekstrom AD, Braskie MN, Thompson PM, Small GW (2008). Reduced cortical thickness in hippocampal subregions among cognitively normal apolipoprotein e e4 carriers. Neuroimage..

[18] Thambisetty M, Wan J, Carass A, An Y, Prince JL, Resnick SM (2010). Longitudinal changes in cortical thickness associated with normal aging. Neuroimage..

[19] Jang H, Kim W, Cho J, Sohn J, Noh J, Seo G (2021). Cohort profile: the Environmental-Pollution-Induced Neurological EFfects (EPINEF) study: a multicenter cohort study of Korean adults. Epidemiol Health..

[20] Ahn HJ, Chin J, Park A, Lee BH, Suh MK, Seo SW (2010). Seoul Neuropsychological Screening Battery-dementia version (SNSB-D): a useful tool for assessing and monitoring cognitive impairments in dementia patients. J Korean Med Sci..

[21] James PA, Oparil S, Carter BL, Cushman WC, Dennison-Himmelfarb C, Handler J (2014). 2014 evidence-based guideline for the management of high blood pressure in adults: report from the panel members appointed to the Eighth Joint National Committee (JNC 8). JAMA..

[22] WHO Expert Consertation (2004). Appropriate body-mass index for Asian populations and its implications for policy and intervention strategies. Lancet.

[23] Benjamini Y, Hochberg Y (1995). Controlling the false discovery rate: a practical and powerful approach to multiple testing. J Royal Stat Soc B Methodol..

[24] Holm S (1979). A Simple Sequentially Rejective Multiple Test Procedure. Scand J Stat..

[25] Wang J, Li T, Sabel BA, Chen Z, Wen H, Li J (2016). Structural brain alterations in primary open angle glaucoma: a 3 T MRI study. Sci Rep..

[26] Frezzotti P, Giorgio A, Toto F, De Leucio A, De Stefano N (2016). Early changes of brain connectivity in primary open angle glaucoma. Hum Brain Mapp..

[27] Catani M, Dell’acqua F, Thiebaut de Schotten M. (2013). A revised limbic system model for memory, emotion and behaviour. Neurosci Biobehav Rev..

[28] LeDoux JE (1994). Emotion, memory and the brain. Sci Am..

[29] Belamkar AV, Mansukhani SA, Savica R, Spiegel MR, Hodge DO, Sit AJ (2021). Incidence of Dementia in Patients With Open-angle Glaucoma: A Population-based Study. J Glaucoma..

[30] Lin IC, Wang YH, Wang TJ, Wang IJ, Shen YD, Chi NF (2014). Glaucoma, Alzheimer’s disease, and Parkinson’s disease: an 8-year population-based follow-up study. PLoS One..

[31] Rezapour J, Nickels S, Schuster AK, Michal M, Munzel T, Wild PS (2018). Prevalence of depression and anxiety among participants with glaucoma in a population-based cohort study: The Gutenberg Health Study. BMC Ophthalmol..

[32] Shin DY, Jung KI, Park HYL, Park CK (2021). The effect of anxiety and depression on progression of glaucoma. Sci Rep..

[33] Zhang X, Olson DJ, Le P, Lin FC, Fleischman D, Davis RM (2017). The Association Between Glaucoma, Anxiety, and Depression in a Large Population. Am J Ophthalmol..

[34] Zikou AK, Kitsos G, Tzarouchi LC, Astrakas L, Alexiou GA, Argyropoulou MI (2012). Voxel-based morphometry and diffusion tensor imaging of the optic pathway in primary open-angle glaucoma: a preliminary study. AJNR Am J Neuroradiol..

[35] Williams AL, Lackey J, Wizov SS, Chia TM, Gatla S, Moster ML (2013). Evidence for widespread structural brain changes in glaucoma: a preliminary voxel-based MRI study. Invest Ophthalmol Vis Sci..

[36] Guo L, Salt TE, Luong V, Wood N, Cheung W, Maass A (2007). Targeting amyloid-beta in glaucoma treatment. Proc Natl Acad Sci U S A..

[37] Gasparini L, Crowther RA, Martin KR, Berg N, Coleman M, Goedert M (2011). Tau inclusions in retinal ganglion cells of human P301S tau transgenic mice: effects on axonal viability. Neurobiol Aging..

[38] Giorgio A, Zhang J, Costantino F, De Stefano N, Frezzotti P (2018). Diffuse brain damage in normal tension glaucoma. Hum Brain Mapp..

[39] Zhao D, Cho J, Kim MH, Friedman DS, Guallar E (2015). Diabetes, fasting glucose, and the risk of glaucoma: a meta-analysis. Ophthalmology..

[40] Zhou M, Wang W, Huang W, Zhang X (2014). Diabetes mellitus as a risk factor for open-angle glaucoma: a systematic review and meta-analysis. PLoS One..

