# Supplementary material for: Reduced brain subcortical volumes in patients with glaucoma: a pilot neuroimaging study using the region-of-interest-based approach
Source: BMC Neurol. 2022 Jul 25;22:277. doi: 10.1186/s12883-022-02807-x (PMC9310417; doi:10.1186/s12883-022-02807-x)
Supplement: Supplementary file 1 — Additional file 1: Supplementary Table 1. Cortical thickness and subcortical volume in individuals with glaucoma compared to age, sex, and educational level-matched individuals without glaucoma (corrected for multiple comparisons using the Bonferroni-Holm method). Supplementary Table 2. Association of glaucoma with cortical thickness and subcortical volume, stratified by history of hypertension. Supplementary Table 3. Association of glaucoma with cortical thickness and subcortical volume, stratified by history of diabetes mellitus. [file 12883_2022_2807_MOESM1_ESM.docx]

**Supplementary material**

**Supplementary Table 1. Cortical thickness and subcortical volume in individuals with glaucoma compared to age, sex, and educational level-matched individuals without glaucoma (corrected for multiple comparisons using the Bonferroni-Holm method)**

|  | | Unadjusted model | | | Adjusted model | | |
| --- | --- | --- | --- | --- | --- | --- | --- |
|  |  | Beta | Standard error | *p | Beta | Standard error | *p |
| Cortical thickness (mm) | Global | -0.020 | 0.012 | 0.51 | -0.011 | 0.014 | 1.0 |
|  | Frontal lobe | -0.015 | 0.015 | 0.98 | -0.007 | 0.018 | 1.0 |
|  | Parietal lobe | -0.035 | 0.015 | 0.12 | -0.021 | 0.017 | 1.0 |
|  | Temporal lobe | -0.024 | 0.015 | 0.51 | -0.016 | 0.018 | 1.0 |
|  | Occipital lobe | -0.040 | 0.013 | 0.014 | -0.016 | 0.015 | 1.0 |
|  | Cingulate | -0.011 | 0.018 | 1.0 | -0.003 | 0.022 | 1.0 |
|  | Insula | 0.004 | 0.021 | 1.0 | 0.002 | 0.024 | 1.0 |
| Subcortical volume (mm^3^) | Thalamus | -300.45 | 73.97 | 0.007 | -212.81 | 88.03 | 0.064 |
|  | Caudate | -187.61 | 63.20 | 0.015 | -170.03 | 73.73 | 0.064 |
|  | Putamen | -126.91 | 63.99 | 0.094 | -151.41 | 75.03 | 0.088 |
|  | Pallidum | -87.45 | 26.36 | 0.007 | -103.59 | 31.26 | 0.007 |
|  | Hippocampus | -110.42 | 47.58 | 0.060 | -141.37 | 55.94 | 0.055 |
|  | Amygdala | -75.44 | 26.58 | 0.020 | -87.94 | 31.42 | 0.030 |
|  | Nucleus accumbens | -16.32 | 10.17 | 0.12 | -20.44 | 11.60 | 0.088 |

*Footnotes*. The adjusted model included the history of disease (hypertension, angina or myocardial infarction, and diabetes), lifestyle factors (smoking status and current alcohol status), marital status, systolic blood pressure, diastolic blood pressure, body mass index, total cholesterol level, fasting blood glucose level, and intracranial volume. P-values were corrected for multiple comparisons using the Bonferroni-Holm method.

**Supplementary Table 2. Association of glaucoma with cortical thickness and subcortical volume, stratified by history of hypertension**

|  | | Individuals with hypertension  (N= 98) | Individuals without hypertension  (N=94) |  |
| --- | --- | --- | --- | --- |
|  |  | Beta (standard errors) | Beta (standard errors) | p value for interaction |
| Cortical thickness (mm) | Global | 0.006 (0.019) | -0.038 (0.023) | 0.15 |
|  | Frontal lobe | 0.003 (0.023) | -0.031 (0.033) | 0.38 |
|  | Parietal lobe | -0.002 (0.022) | -0.023 (0.031) | 0.57 |
|  | Temporal lobe | -0.008 (0.026) | -0.046 (0.030) | 0.33 |
|  | Occipital lobe | -0.012 (0.018) | -0.002 (0.026) | 0.76 |
|  | Cingulate | 0.016 (0.029) | -0.066 (0.039) | 0.10 |
|  | Insula | 0.026 (0.032) | -0.030 (0.043) | 0.30 |
| Subcortical volume (mm^3^) | Thalamus | -338.03 (89.25) | 14.23 (188.06) | 0.09 |
|  | Caudate | -85.34 (86.01) | -323.60 (149.58) | 0.17 |
|  | Putamen | -96.69 (91.50) | -287.35 (145.33) | 0.27 |
|  | Pallidum | -111.64 (36.25) | -68.83 (56.62) | 0.52 |
|  | Hippocampus | -200.88 (67.21) | -24.80 (105.09) | 0.16 |
|  | Amygdala | -91.57 (42.00) | -83.19 (59.44) | 0.91 |
|  | Nucleus accumbens | -15.28 (14.20) | -44.77 (21.13) | 0.25 |

*Footnotes*. The adjusted model included the history of disease (angina or myocardial infarction, and diabetes), lifestyle factors (smoking status and current alcohol status), marital status, systolic blood pressure, diastolic blood pressure, body mass index, total cholesterol level, fasting blood glucose level, and intracranial volume.

**Supplementary Table 3. Association of glaucoma with cortical thickness and subcortical volume, stratified by history of diabetes mellitus**

|  | | Individuals with diabetes  (N=50) | Individuals without diabetes  (N=142) |  |
| --- | --- | --- | --- | --- |
|  |  | Beta (standard errors) | Beta (standard errors) | p value for interaction |
| Cortical thickness (mm) | Global | 0.004 (0.038) | -0.021 (0.018) | 0.18 |
|  | Frontal lobe | 0.003 (0.048) | -0.015 (0.024) | 0.46 |
|  | Parietal lobe | 0.001 (0.041) | -0.022 (0.023) | 0.49 |
|  | Temporal lobe | 0.003 (0.042) | -0.028 (0.024) | 0.21 |
|  | Occipital lobe | -0.041 (0.040) | -0.008 (0.018) | 0.45 |
|  | Cingulate | 0.053 (0.061) | -0.031 (0.027) | 0.21 |
|  | Insula | 0.152 (0.051) | -0.012 (0.032) | 0.01 |
| Subcortical volume (mm^3^) | Thalamus | -384.53 (184.29) | -164.76 (125.07) | 0.32 |
|  | Caudate | -142.72 (163.33) | -206.63 (100.50) | 0.74 |
|  | Putamen | -146.70 (200.57) | -224.09 (99.09) | 0.73 |
|  | Pallidum | -138.20 (92.96) | -103.10 (40.23) | 0.73 |
|  | Hippocampus | -308.15 (131.85) | -140.96 (73.31) | 0.27 |
|  | Amygdala | -139.40 (72.50) | -92.44 (43.33) | 0.58 |
|  | Nucleus accumbens | 22.01 (27.48) | -37.17 (15.92) | 0.06 |

*Footnotes*. The adjusted model included the history of disease (hypertension, and angina or myocardial infarction), lifestyle factors (smoking status and current alcohol status), marital status, systolic blood pressure, diastolic blood pressure, body mass index, total cholesterol level, fasting blood glucose level, and intracranial volume.
